# Cryo-EM Map–Based Model Validation Using the False Discovery Rate Approach

**DOI:** 10.3389/fmolb.2021.652530

**Published:** 2021-05-18

**Authors:** Mateusz Olek, Agnel Praveen Joseph

**Affiliations:** ^1^ Department of Chemistry, University of York, York, United Kingdom; ^2^ Electron BioImaging Center, Rutherford Appleton Laboratory, Didcot, United Kingdom; ^3^ Scientific Computing Department, Science and Technology Facilities Council, Research Complex at Harwell, Didcot, United Kingdom

**Keywords:** cryo-EM, model validation, FDR map, CCP-EM, automated model building

## Abstract

Significant technological developments and increasing scientific interest in cryogenic electron microscopy (cryo-EM) has resulted in a rapid increase in the amount of data generated by these experiments and the derived atomic models. Robust measures for the validation of 3D reconstructions and atomic models are essential for appropriate interpretation of the data. The resolution of data and availability of software tools that work across a range of resolutions often limit the quality of derived models. Hence, the final atomic model is often incomplete or contains regions where atomic positions are less reliable or incorrectly built. Extensive manual pruning and local adjustments or rebuilding are usually required to address these issues. The presented research introduces a software tool for the validation of the backbone trace of atomic models built in the cryo-EM density maps. In this study, we use the false discovery rate analysis, which can be used to segregate molecular signals from the background. Each atomic position in the model can be associated with an FDR backbone validation score, which can be used to identify potential mistraced residues. We demonstrate that the proposed validation score is complementary to existing validation metrics and is useful especially in cases where the model is built in the maps having varying local resolution. We also discuss the application of the score for automated pruning of atomic models built *ab-initio* during the iterative model building process in Buccaneer. We have implemented this score in the CCP-EM software suite.

## Introduction

Improvements in cryo-EM data collection and processing techniques in recent years have enabled structure determination at near-atomic resolutions (Subramaniam, 2019). For structure interpretation, a number of tools for *ab-initio* model building have been developed and used in recent years (Hoh et al., 2020; Terwilliger et al., 2020; Pfab et al., 2021; Lawson et al., 2021). Despite the resolution revolution, the majority of maps (92%) deposited in the EM Data Bank (https://www.ebi.ac.uk/pdbe/emdb/) are at resolutions worse than 3 Å, and the average resolution of maps this year is around 5 Å (https://www.ebi.ac.uk/pdbe/emdb/statistics_sp_res.html/). Moreover, some local areas in the cryo-EM map can be poorly resolved. These issues may result in some parts of the derived atomic model being incorrectly built or traced into background noise. Model validation tools that are based on the analysis of stereochemical properties of the atomic model, such as MolProbity (Williams et al., 2018), CaBLAM (Prisant et al., 2020), or Ramachandran plots, detect potential issues with the geometry of the model. The users can inspect the possible incorrect regions of the model and attempt to fix these in interactive visualization tools like Coot (Emsley et al., 2010).

Another set of validation tools evaluate the agreement of the atomic model with the cryo-EM map. Some of these scores can estimate the agreement of each residue against the area of the map covered by the residue. The agreement is either quantified as Manders’ overlap coefficient in SMOC (Joseph et al., 2016), real space cross-correlation coefficient in PHENIX local CCC (Afonine et al., 2018), or a score of atomic resolvability in MapQ (Pintilie 2020). One of the observations from the recent model challenge is that the absolute values of some of these metrics are sensitive to the map resolution (Lawson et al., 2021). One reason is the underlying sensitivity of the metric toward differences in the shape of map distributions at different resolutions. Another reason is the fact that the synthetic map calculation from the model may not be optimal to represent experimental data at different resolutions.

The recently introduced FSC-Q score allows us to assess the local agreement of a model with the cryo-EM density map, and is normalized to account for local resolution variation (Ramírez-Aportela et al., 2021). The map-model local Fourier shell correlation (FSC) is normalized with respect to the local FSC obtained from the halfmaps. The FSC-Q score is calculated as the difference between these two and the values fluctuates around 0. A threshold of +/− 0.5 is recommended to detect poorly fitted atoms. Although the FSC-Q calculation is not directly affected by the B-factor values used for map sharpening, the mask applied can have an effect in the local FSC calculation.

MapQ scores atoms in the residues by comparing the distance-dependent map value fall-off against a Gaussian-like reference derived from a map of apoferritin resolved at 1.54 Å and an associated well-fitted atomic model. The Q-score is calculated as a correlation between the map values and the reference Gaussian. Values close to 1 indicate that the atom is well resolved (Pintilie, 2020).

Metrics such as the atom-inclusion score (Lagerstedt et al., 2013), implemented as part of EMDB validation analysis, identify atoms in the model that are outside a selected map contour. The score is hence very sensitive to the choice of map contour, which is often subjective. Also, in cases where the local resolution varies across the map, a single contour may not be optimal to cover the entire molecular volume without including the background noise.

The resolution of cryo-EM maps may vary as a result of molecular flexibility, partial occupancy, non-uniform particle orientation, and other factors associated with the reconstruction process. Often, the resolution is better in the core and it gradually worsens toward the edges or other flexible parts of the molecular assembly. The statistical analysis used in the false discovery rate (FDR) approach allows associating confidence in distinguishing molecular signals from the background and detecting weak features in the map based on the statistical significance estimate. The FDR calculation (Beckers et al., 2019) generates confidence maps with values at each voxel reflecting the fraction of voxels expected to contain molecular signals at this threshold (the voxel value). The 1% FDR threshold (confidence map threshold of 0.99) was demonstrated to reliably discriminate voxels associated with the molecular volume from the background noise over a wide resolution range, including maps at near-atomic resolutions to 6.8 Å and the subtomogram averages in the resolution range 7–90 Å.

In this study, we present a tool for validating the backbone trace of an atomic model by estimating the confidence that the backbone atoms are in the molecular volume rather than the background. Each residue in the model are assigned scores based on the confidence map calculated using the FDR approach. We demonstrate the utility of the approach to detect mistraced residues, using datasets from the EMDB model challenges 2015/16 and 2019, and compare it against other metrics used in the field for estimating local fit to maps.

This procedure is also useful for pruning mistraced regions of the model generated by *ab-initio* modeling tools like Buccaneer (Hoh et al., 2020). Especially at areas of the map with resolution worse than 3.5 Å, it is not uncommon that the chain may be mistraced into the background. Also, Buccaneer often traces a few polypeptide fragments in the background areas with noisy features or artifacts from map reconstruction and postprocessing. These fragments are not connected with the main chains of the model and usually are only of a few residues long. Currently, there is no automated tool to locate and remove mistraced residues. Where possible, the pruned models can then be extended with one of the automated model building tools or rebuilt in an interactive tool like Coot.

Initial results indicate that our approach is effective in detecting mistraced regions of the model and for automated pruning of models as part of Buccaneer. The FDR backbone validation score assesses whether the backbone coordinates are within the molecular volume and is complementary to existing validation tools that either assess the model quality or evaluate agreement with the map. The described tool is implemented and available as a part of the CCP-EM (Burnley et al., 2017) software suite.

## Methods

The FDR backbone validation method assesses positions of the atoms in the input model based on the confidence map derived using the FDR approach (Beckers et al., 2019). For the confidence map calculation, a processed/sharpened but unmasked map is preferred. Masked maps that exclude the majority of solvent background are not useful for confidence map calculations. The procedure estimates the background noise distribution from four density cubes placed outside of the particle volume in the x, y, and z central axes by default. Each voxel of the map is then compared against the background estimate to detect significant deviations and a *p*-value is associated to quantify significance. To account for the number of voxels and their dependencies, the *p*-values are further adjusted using false discovery rate (Benjamini and Yekutieli, 2001). Each voxel is assigned with an FDR-adjusted significance score between 0 and 1, 0 refers to noise only and 1 to a clear molecular signal. A score of 0.99 indicates that a maximum of 1% of voxels (1% FDR) is expected to be background noise, beyond this threshold.

We use the following steps to calculate the FDR backbone score:

(1)The minimal input for the FDR-validation is the pdb or cif/mmcif format file of the atomic model and the confidence map calculated using the FDR control approach. The confidence map can be calculated using the “confidence map” implementation in the CCP-EM software suite or using a standalone installation from the source (https://git.embl.de/mbeckers/FDRthresholding).
(2)The input model coordinates are extracted and mapped onto the confidence map grid by associating the voxel(s) around the atomic coordinate (within 1 Å).
(3)Each atom of the model is then associated with the corresponding map value from the confidence map. In the default mode, the FDR backbone score of each residue is calculated as an average of map values at the coordinates of the C-alpha, C, and N atoms. We use this approach primarily to detect mistraced residues based on the positions of backbone coordinates in the map. We exclude the backbone carbonyl oxygen as they are often associated with weak map information at resolutions worse than 3 Å. This approach can be used to detect misplacement of the side chains as well, although missing map data at the ends of acidic and highly flexible side chains can lead to false detections. For nucleic acids, the average score is calculated based on the C1′, C2′, C3′, C4′, C5′, O3′, O4′, O5′, and P atoms positions. For ligands and waters, all of the atoms are taken into consideration. The users can also choose an optional validation mode based solely on the Cα positions of the residues and C1′ for nucleic acids. This mode is useful with models with only Cα atoms, usually built in low-resolution cryo-EM maps.
(4)Additionally, this tool offers an option to prune the atomic model, which can be used to automatically remove the residues with a score lower than 0.9 as well as the preceding and following residues. A model pruned this way can be used in the next stages of the iterative model building procedures, where the missing segments can be extended or rebuilt. This is useful when dealing with the *ab-initio* models from the automated model building tools. In some cases, particularly when building in areas of the map with a local resolution worse than 3.5 Å, parts of the chains can be traced into the background.
(5)As an output, we provide a CSV format file containing the list of residues with the associated FDR backbone scores. The models after pruning will have the low scoring residues removed. They are saved in the selected folder with the original model names with a suffix “pruned” added depending on the mode used. We also provide an attribute file that can be used to associate the FDR score for each residue in the atomic model in UCSF Chimera (Pettersen et al., 2004). The model can then be colored using the FDR score attribute to identify areas with low scores.

The FDR backbone validation tool is written in Python 3. To handle the I/O model files in pdb and cif/mmcif format the GEMMI package (Wojdyr, 2017) is used. The map files are processed with the mrcfile python package (Palmer, 2016). The tool also requires NumPy (tested with v1.16.2 (NumPy v1.16 Manual’ 2019)). The GUI implementation with CCP-EM software suite was done using PyQt (‘PyQt 4.9.4 Reference Guide’ 2011).

In this study, we compare the FDR backbone validation approach against other metrics for estimating local fit to maps. For a fair comparison, the other metrics were also calculated only on the backbone atoms of the models. The map deposited in EMDB as a “primary” map was used for the analysis, the FSC-Q score calculation also requires the half-maps.

The Q-score values for the backbone atoms were calculated using the MapQ plugin (v1.6) for UCSF Chimera, at the resolution reported for the deposited primary map.

The FSC-Q score was calculated using the tool (validate fsc-q) integrated in the Scipion v3.0.7-Eugenius. The FSC-Q value for the backbone is calculated as an average for the C, N, and Cα atoms.

The SMOC and SCCC scores were calculated using the score_smoc.py script available from TEMPy1 in CCP-EM v1.5, for the minimal backbone of the models (C, N, and Cα atoms). The script was run with the “-distance” mode option which uses distance from the atoms for identifying voxel-covered. SMOC estimates the Manders’ overlap coefficient while SCCC calculates the cross correlation coefficient.

The PHENIX map/model CCC (v1.18.2) scores were calculated on the models obtained from 10 cycles of atomic B-factor refinement with REFMAC5 (Murshudov et al., 2011) (using the keyword option “refi bonly”). This was done to ensure that the atomic B-factors are refined as PHENIX uses the atomic B-factors as part of the map calculation from the atomic model.

The box size of the input map was trimmed wherever possible to improve the speed of the computations. The FDR-validation requires a sharpened but unmasked map, with the background features present in order to estimate the noise distribution.

## Results

To demonstrate the application of our approach, we used the following examples, the majority of which are models submitted to the EMDB model challenges for target maps resolved at a range of resolutions. In each case, we compare the FDR backbone scores against other metrics that estimate local fit to map. Using a set of residues detected as “mistraced” by the FDR backbone score, we assess agreement with other scores and also highlight cases where there is a disagreement. We use the reference model from the model challenge to compare the backbone conformation and fit to map. Please note that the reference model does not always have the best fit to map for all residues in the model, and often several of the models submitted to the challenge have a better fit (Lawson et al., 2021). In some cases, there are obvious backbone misfits in the reference model, as discussed below. In such cases, we also compare the model of interest against other models reported with a higher rank higher in the model challenge based on a number of validation metrics (https://model-compare.emdataresource.org/).

### Alcohol Dehydrogenase (2.9 Å, Target T0104)

**FIGURE 1:**
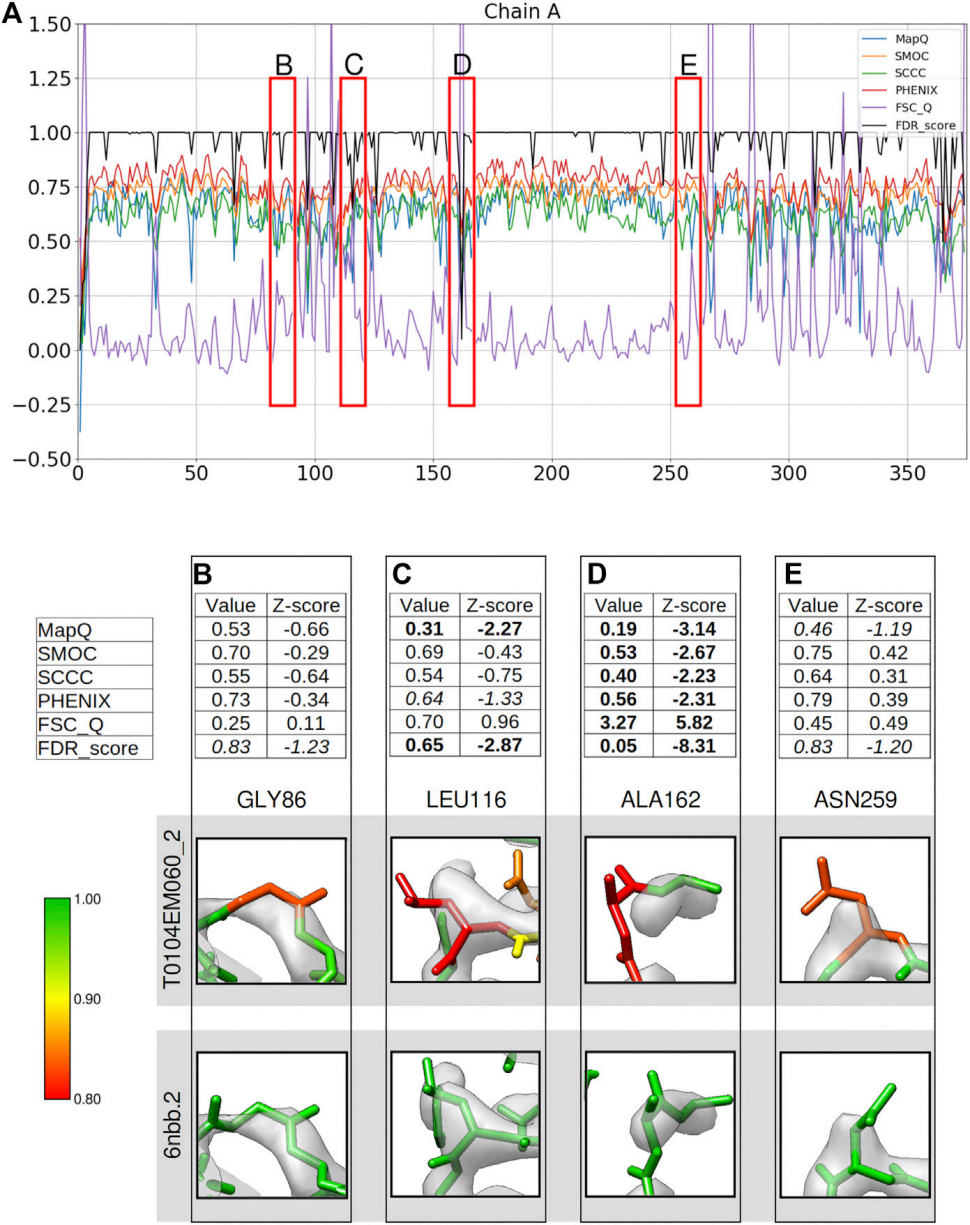
Comparison of local assessment metrics for the atomic model of alcohol dehydrogenase (color bar shows the correspondence with the FDR scores assigned, residues in red have FDR scores around 0.8 or worse, yellow around 0.9, and green around 1.0). The metrics are calculated only for the backbone atoms. **(A)** Per-residue plot of MapQ, SMOC, SCCC, PHENIX, FSC-Q, and FDR backbone scores for the chain A of the atomic model T0104EM060_2 from the EMDB model challenge, the red boxes highlight the residues selected for detailed analysis in the panels below. For Gly 86 **(B)**, Leu 116 **(C)**, Ala 162 **(D),** and Asn 259 **(E)**, the panel shows a table with values of scores obtained with each metric and corresponding Z-scores; the residue fit in the target map (EMD-0406, gray) displayed at the recommended contour level and rendered in UCSF Chimera; and the residue fit in map as modeled in the reference (PDB ID: 6nbb.2).

**FIGURE 2:**
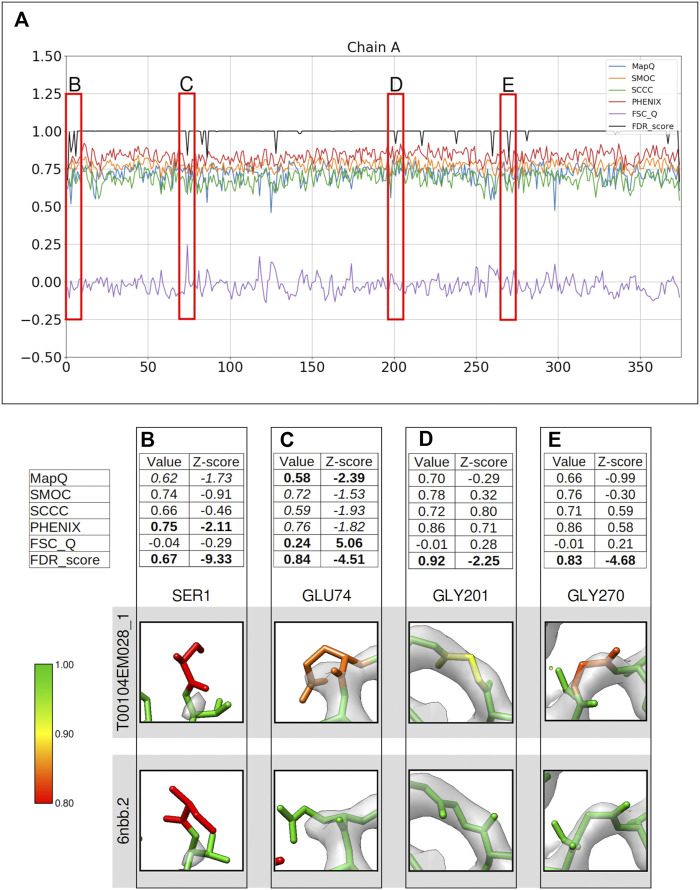
Comparison of metrics for the atomic model of alcohol dehydrogenase (color bar shows the correspondence with the FDR scores assigned, residues in red have FDR scores around 0.8 or worse, yellow around 0.9, and green around 1.0). The metrics are calculated only for the backbone atoms. **(A)** Per-residue plot of the scores from MapQ, SMOC, SCCC, PHENIX, FSC-Q, and FDR backbone score for the chain A of the atomic model T0104EM028_1 from the EMDB model; the red boxes highlight the residues selected for detailed analysis in the panels below. For Ser1 **(B)**, Glu 74 **(C)**, Gly 201 **(D),** and Gly 270 **(E)**, the panel shows a table with values of scores obtained with each metric and corresponding Z-scores; the residue fit in the target map (EMD-0406, grey) displayed at the recommended contour level and rendered in UCSF Chimera; and the residue fit in map as modeled in the reference (PDB ID: 6nbb.2).

We computed per-residue backbone scores based on different metrics for the chain A of model T0104EM060_2 submitted to the Model Challenge 2019 for the target alcohol dehydrogenase map (EMD-0406) resolved at 2.9 Å resolution (Herzik et al. 2019; Figure 1A). We checked residues either associated with lower confidence scores (0.95 or lower) or where the scores disagree in detecting a mistrace, and compared against the reference model used in the model challenge (PDB ID: 6nbb). The reference structure has 10 models representing local conformational variability. We chose the second model (6nbb.2) for our analysis as it has a relatively better fit with the map when inspected in UCSF Chimera, for cases we discuss in Figures 1, 2, especially at the N-terminus (Figure 2B). The metrics used for comparison includes Q-score, SMOC, SCCC, PHENIX local CCC, and FSC-Q, calculated only for the backbone atoms (see Methods). The residues highlighted in red boxes in Figure 1A indicate some of the regions where the scores differ. We provide a detailed analysis of these residues. Figures 1B–E show a table with the values of each metric and corresponding Z-scores, along with a snapshot of the residue colored by the FDR backbone score. Also, the corresponding view of the residue from the reference model 6nbb.2 ((Herzik et al., 2019), model 2) is provided. The models are overlaid with the deposited cryoEM density map EMD-0406 (Herzik et al., 2019) and rendered at the author-recommended contour level 0.02 (0.6σ).

Compared to a few other models submitted to the model challenge, the model T0104EM060_2 is ranked lower by the validation metrics used in the challenge (https://model-compare.emdataresource.org/2019/cgi-bin/em_multimer_results.cgi?target_map=T0104emd_0406). Plot of per-residue backbone scores for the chain A (Figure 1A), also shows that many residues in this model are associated with lower scores (drops in the plot). We investigated a few residues including cases where the metrics disagree. Gly86 is highlighted as a potential mistrace by the FDR backbone score of 0.83 (Figure 1B). The residue in the reference model has a high FDR score (0.991) and the backbone shows better fit to map with a different conformation involving a shift and differences in backbone dihedrals. Z-scores computed for different metrics reflect that none of the other scores identify this mistrace with any significance (absolute value of Z-scores < 1). Note that the Z-scores for FDR backbone assessment are less reliable, especially because the majority of the residues often have a score of 1.0 and the distribution is not close to normal. We recommend using the absolute values of this score to detect potential mistraces.

Figure 1C shows Leu116 associated with a low FDR backbone score of 0.65. It can be seen that the backbone Cα and C are out of the map at the recommended contour level. In comparison, the reference model shows a better fit of backbone atoms. The mistrace is also detected by the MapQ score (0.31, Z-score −2.27), while PHENIX-CCC (0.63, Z-score −1.33) and FSC-Q (0.70, Z-score 0.96) have lower scores but associated with relatively low significance (Z-scores of −1.33 and 0.96, respectively).

Another residue Ala162 is located at a relatively disordered or low-resolution area of the map (Figure 1D), and the backbone is partly out of the map contour when compared to the reference model. All the scores identify the mistrace with significance (absolute Z-scores > 2.0), and the residue is associated with a low FDR backbone score of 0.05. Even though the map at the recommended contour level does not fully support the backbone, the reference model shows a better fit and has an FDR backbone score of 0.978. This reflects that the FDR backbone score detects voxels covering molecular volume even in the low resolution areas of the map.

Figure 1E shows Asn259 associated with an FDR backbone score of 0.83 with part of the backbone outside the contoured map. The reference model shows a better fitted backbone conformation (Figure 1E). MapQ also points to the potential mistrace in the submitted model with a Q-score of 0.46 although with a less significant Z-score of −1.19. The other metrics fail to identify this issue with the backbone fit. Hence, in comparison to other metrics tested in this study, the FDR backbone score detects cases of mistrace where one or more backbone atoms are displaced into background noise.

Figure 2 presents a similar analysis of the model T0104EM028_1 submitted to the same target map. Ser1 at the N-terminus of chain A is associated with an FDR backbone score of 0.67, clearly indicating a potential mistrace. Ser1 is associated with a disordered area of the map with no prominent map information at the recommended contour (Figure 2B). PHENIX_CC (0.75, Z-score −2.11) and MapQ (0.62, Z-score −1.73) scores also suggest poor agreement with data. The map trace is more obvious at a lower contour level (Supplementary Figure S1), and the terminal N atom is outside the map even at this level. Hence, there is less confidence associated with the backbone atom positions and this is also highlighted by PHENIX_CC and MapQ scores.

Figure 2C highlights Glu74 with both backbone and side chain atoms out of the recommended contour. The residue, as modeled in the reference, shows better fit with backbone atoms (and most of the side chain) inside the recommended contour. The lack of map information for the end of side chain is a common trait observed in cryo-EM maps for negatively charged side chains. FSC-Q and MapQ indicate a backbone mistrace with Z-score values less than −2.0 (>2.0 in case of the FSC-Q score). The other metrics also highlight this, although with a relatively lower significance (Z-score < −1.5).

Gly201 is associated with an FDR validation score of 0.92. Other scores do not seem to indicate mistrace with any significance (all Z-scores were between −1 and 1) (Figure 2D). This residue has a different backbone conformation in the reference model and is associated with an FDR backbone score of 1.0. The backbone has a better fit in the reference with all atoms except carbonyl oxygen inside the recommended contour. Another case where only the FDR-validation score detects a mistrace of backbone is Gly270, where the reference model shows a better fit with the map with a slight shift in atom positions (Figure 2E). The backbone residue shifted outside of the map density is presented in Figure 2E. These cases highlight that the FDR backbone score can work in complementarity to the scores that quantify agreement with the map.

The structure of alcohol dehydrogenase has zinc ions bound but the ions are not modeled in all of the structures submitted to the model challenge. Supplementary Figure S2A presents a comparison of two models submitted (Model Challenge IDs: T0104EM010_1 on the left panel and T0104EM028_1 on the center) where the zinc atoms are modeled, along with the reference model (PDB ID: 6nbb) on the right. In the model T0104EM010_1, the zinc atom is highlighted as a potential misfit based on our approach, and no obvious map data can be seen at this position. It can be seen that the ligands in both the model T0104EM028_1 and the reference structure are placed in a position justified by map density and supported by the higher FDR backbone scores. It is worth mentioning that many of the automated model building software do not support ligand fitting, and therefore this is often done interactively. The presented validation technique can be useful for validating the modeled ligands in cryo-EM maps.

### T20s Proteasome (2.8 Å)

**FIGURE 3:**
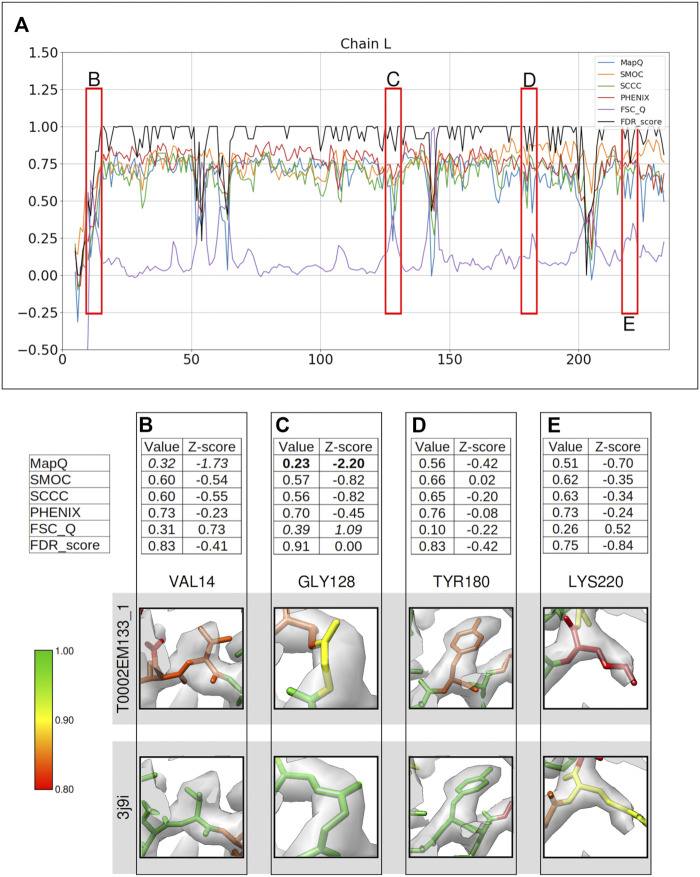
Comparison of metrics for the atomic model of T20s proteasome, calculated only for the backbone atoms. **(A)** Comparison of the per-residue scores from MapQ, SMOC, SCCC, PHENIX, FSC-Q, and FDR backbone score for the chain L of the atomic model T0002EM133_1 from the EMDB model; the red boxes highlight the residues selected for detailed analysis in the panels below. For Val 14 **(B)**, Gly 128 **(C)**, Tyr 180 **(D),** and Lys 220 **(E)**, the panel shows a table with values of scores obtained with each metric and corresponding Z-scores; the residue fit in the target map (EMD-6287, grey) displayed at the recommended contour level 0.025 (3.3σ) and rendered in UCSF Chimera; and the residue fit in map as modeled in the reference (PDB ID: 3j9i).

Another set of models used for the evaluation of the FDR backbone validation approach were chosen from those submitted to the model challenge for the target map of the T20s proteasome (EMD-6287), resolved at 2.8 Å resolution. Figure 3A presents the comparison of scores from difference metrics obtained for the chain L of the model T0002EM133_1. Again, the areas where the scores disagree were inspected closely.

Several residues in this model are associated with lower score values as evaluated by different metrics (Figures 3B–E). In this case, the Z-scores are less meaningful as the distribution of scores is likely to deviate significantly from normal because of the presence of several low-scoring residues (outliers). Therefore, we considered a less stringent absolute Z-score cutoff of 1.0 to associate significance to the scores. Again, as the FDR scores do not follow a normal distribution (often many residues have a score of 1.0 and a few scoring lower), the Z-scores are less useful. We recommend using the absolute FDR scores to detect potential mistraces.

Val14 is associated with a low FDR score of 0.83, also supported by a lower MapQ score of 0.32 (Z-score −1.73) (Figure 3B). The backbone N atom is out of the map contoured at the recommended level. The reference model (PDB ID: 3j9i) shows a better fit and has an FDR score of 1.0. Hence, the FDR backbone score and MapQ detect the mistrace with a greater significance compared to other metrics.

Figure 3C shows Gly128 which has been scored lower by MapQ (0.23, Z-score: −2.20), and FSC-Q has a score of 0.39, although with a relatively less significant Z-score of 1.09. The backbone of this residue is associated with an FDR backbone score of 0.91. Visual inspection of the backbone shows that the Cα and carbonyl C atoms are partly out of the contoured map. The reference model shows a better fit of this residue with a higher FDR score of 0.99.

Tyr180 is assigned a low FDR backbone score of 0.83 (Figure 3D), and the other scores do not highlight a backbone misplacement with all absolute Z-score values less than 0.5. The backbone N atom of the modeled residue is partly outside the contoured map. The reference model (chain B) shows a better backbone and a side chain fit and has an FDR backbone score of 0.99. Hence, the FDR score detects backbone misplacements compared to other metrics used in this study and is thus effective in identifying mistraced residue backbone.

This is another case where the scores disagree is Lys220 (Figure 3E), which is associated with a low FDR backbone score of 0.75, while the other scores do not highlight a mistrace with significance. The MapQ score is relatively lower with a value of 0.51 (Z-score: −0.70). A closer inspection and comparison with reference suggests that the residue has a better placement in the reference with a shift of backbone atoms accompanied by better positioning of side chain. The residue backbone in the reference was assigned an FDR score of 0.92 and the residues on either side of Lys220 also score low. This highlights the possibility of further improvement of backbone atom placement in this segment of the reference.

### γ-Secretase (3.4 Å, Target T0007)

**FIGURE 4:**
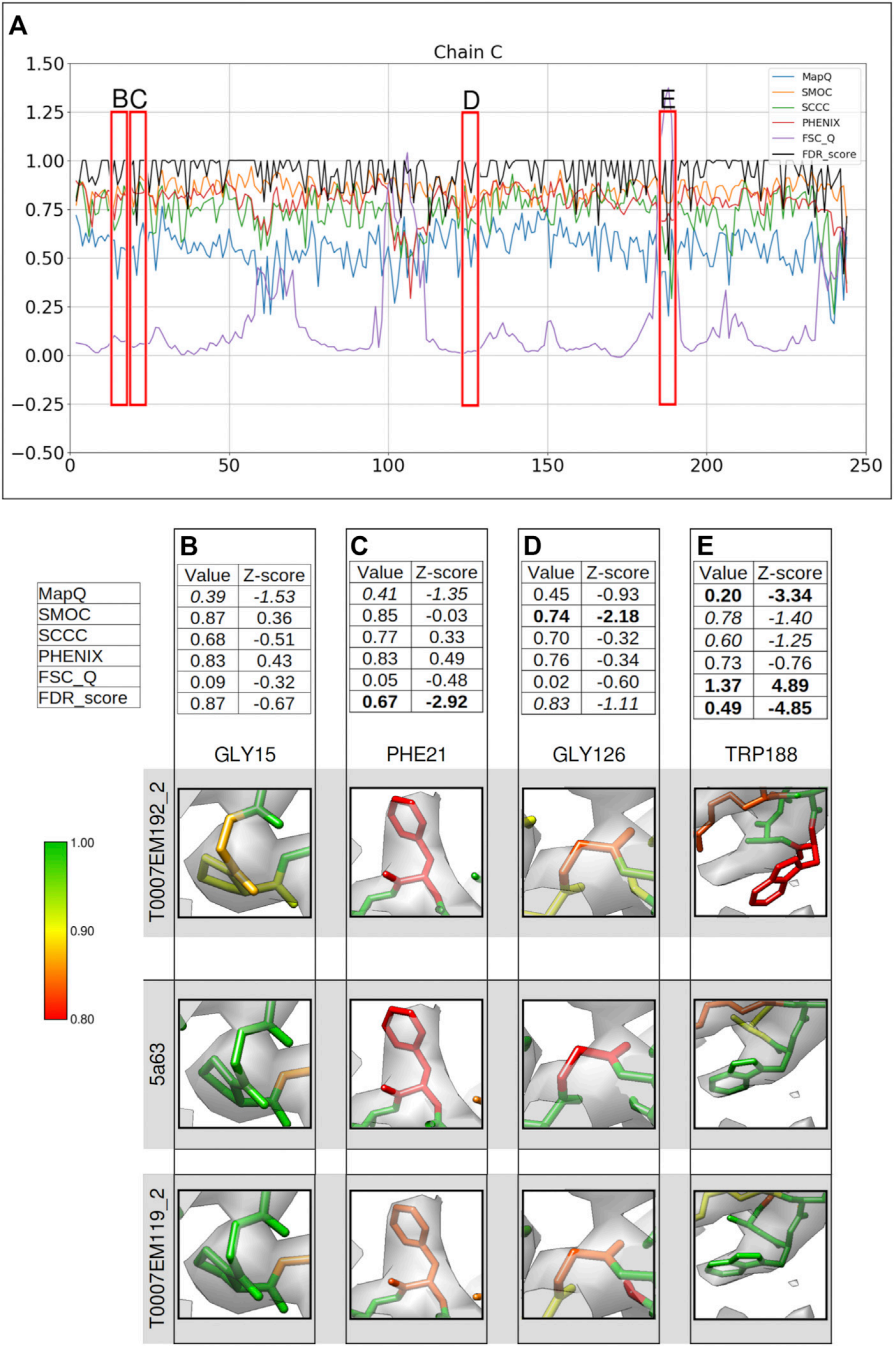
Comparison of metrics for the atomic model of γ-secretase, calculated only for the backbone atoms. **(A)** Comparison of per-residue scores from MapQ, SMOC, SCCC, PHENIX, FSC-Q, and FDR backbone score for the chain C of the atomic model T0007EM192_2 from the EMDB model challenge; the red boxes highlight the residues selected for detailed analysis in the panels below. For Gly 15 **(B)**, Phe 21 **(C)**, Gly 126 **(D),** and Trp 188 **(E)**, the panel shows a table with values of scores obtained with each metric and corresponding Z-scores; the residue fit in the target map (EMD-3061, grey) displayed at the recommended contour level and rendered in UCSF Chimera (first row); the residue fit in map as modeled in the reference (PDB ID: 5a63, center), and the fit of model T0007EM119_2 in the map (last row).

The FDR backbone validation tool was used to assess another model (Model Challenge ID: T0007EM192_2) submitted to the EMDB Model Challenge 2015/2016 (Lawson and Chiu, 2018) for the gamma secretase map EMD-3061, solved at 3.4 Å resolution (Bai et al., 2015). Figure 4 shows the comparison of different scores associated with residues in the chain C of the model. Figures 4B–E provide a closer look into some of the areas of the model where the scores disagree. As discussed below, the reference model (PDB ID: 5a63) does not show a better fit for most of these cases. Hence, we also compared the backbone fit against T0007EM119_2, which is another model submitted to the model challenge for this target and ranked higher than the reference by multiple metrics used in the challenge.

Gly15 is associated with an FDR backbone score of 0.87 (Figure 4B) and MapQ also associates a low score of 0.39 with the backbone (Z-score: −1.53). Other scores do not highlight a mistrace of backbone atoms for this residue. Visual inspection shows that the N and Cα atoms are outside the map at the recommended contour. In the reference model (PDB ID: 5a63), the backbone shows a slight shift of the backbone toward the map volume. In the model T0007EM119_2, which scored higher than the reference in the model challenge, the atoms are shifted well into the map and Gly15 has an FDR score of 1.0. Hence, the slight backbone misplacement is highlighted by FDR and MapQ scores in this case.

Phe21 is also associated with a low FDR validation score of 0.67 and MapQ associates a relatively lower Q-score of 0.41 (Z-score: 1.35) (Figure 4C). The reference model shows similar backbone atom positions but associated with a lower FDR backbone score (0.58). Upon closer inspection of the model at a higher contour level, we find that the carbonyl C atom is out of the map. In the model T0007EM119_2, the residue shows a slightly better fit with the backbone shifted into the map, and has an FDR backbone score of 0.83. Multiple metrics (the FDR score and MapQ) point to a potential backbone misfit and further investigation is required in this case to establish this and check for improvement upon refitting.

Figure 4D shows Gly126 with the Cα atom outside the map at the recommended contour. The modeled residue is detected as potential mistrace with an FDR backbone validation score of 0.83 and a lower SMOC score of 0.74 (Z-score −2.18). Other scores do not highlight this with a significant Z-score. The backbone of Gly126 in the reference model (PDB ID: 5a63) is also partly outside the contoured map and associated with a lower FDR score (0.75). In the model T0007EM119_2, a similar scenario was found where the Cα atom is partly outside the map contour. Both the FDR score and SMOC identify a misfit in this case reflecting a potential for improvement of the backbone fit. In the absence of a good reference fit, further investigation and refitting is required to confirm the backbone misplacement.

Another residue associated with a low FDR backbone score of 0.49 is Trp188 (Figure 4E). The backbone mistrace is evident in this case when compared to the reference structure (PDB ID: 5a63), where Trp188 is better fitted in the map (Figure 4E) and has an FDR backbone score of 1.0. FSC-Q and MapQ scores also detect the backbone misplacement with significant Z-scores. The model T0007EM119_2 also shows a well-fitted backbone with an FDR score of 0.995. Hence, in this case, the FDR score works in complementarity with the metrics that calculate CCC or similar (SMOC).

Supplementary Figure S2B highlights another segment of the model (T0007EM192_2) with a polysaccharide, where the atomic positions in the terminal monosaccharide units have relatively lower FDR scores. The FDR validation score is calculated as an average of scores of the atoms in each unit. These terminal units of the carbohydrate are expected to be more flexible and the range of values of the score reflects this as well, suggesting higher uncertainty of the positions at the edges for being associated with molecular signals. The terminal monosaccharide unit in the reference model (PDB ID: 5a63) is also associated with lower FDR validation scores.

### RNA Polymerase Complex From SARS-CoV-2 (2.5 Å)

**FIGURE 5:**
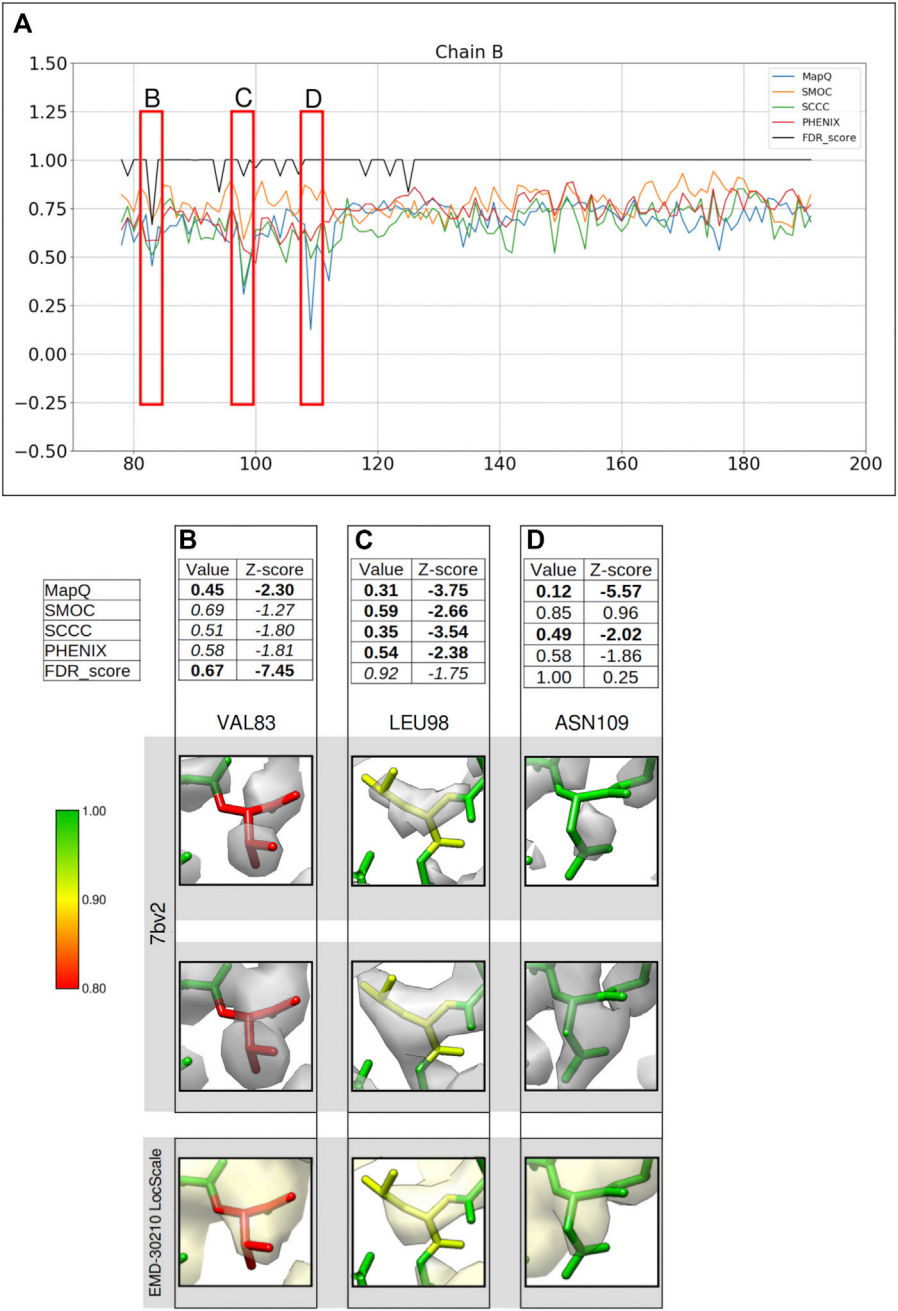
Comparison of backbone validation metrics for the atomic model of the RNA polymerase complex (PDB ID: 7bv2). **(A)** Comparison of the per-residue scores from MapQ, SMOC, SCCC, PHENIX, and FDR backbone score for the chain B of the atomic model, the red boxes highlights the residues selected for detailed analysis in the panels below. For Val 83 **(B)**, Leu 98 **(C)**, and Asn 109 **(D)**, the panel shows a table with values of scores obtained with each metric and corresponding Z-scores; the residue fit in the target map (EMD-30210, grey) displayed at the recommended contour level and rendered in UCSF Chimera; the residues fit in the map rendered at a lower contour level.

We also applied our approach to assess the atomic model (PDB ID: 7bv2) deposited with the recently published structure of RNA polymerase complex (EMD-30210, 2.5 Å) from SARS-CoV-2 virus (Yin et al., 2020). A few residues in the model have lower confidence scores assigned (Figure 5A).

Figure 5 shows a comparison of the validation metrics for residues in the chain B of the model. The FSC-Q score was not calculated for this case as the half maps were not available from EMDB. Figures 5B–D provide insights into selected regions of the model, fitted in the map contoured at the recommended level 0.058 (4.3σ). At this contour the map data corresponding to most of the backbone of low-scoring residues at the N-terminus is disconnected, possibly indicating relatively lower local resolutions. We also assessed the backbone atom placement at a lower contour level (0.035) (Figures 5B–D, second row). To check whether the disconnected map data is due to local over-sharpening (often resulting from a global sharpening factor applied to the map), we calculated a locally sharpened map using LocScale (Jakobi et al., 2017) implemented in the CCP-EM software suite (Figures 5B–D, last row).

Figure 5B shows Val83 highlighted as a potential mistrace by the FDR score with a value of 0.67 and MapQ (0.45, Z-score −2.30). The poor quality of fit is also indicated by other metrics including SMOC (0.69, Z-score −1.27), SCCC (0.51, Z-score −1.80), and PHENIX (0.58, Z-score −1.81). It can be seen that this residue backbone is not fully supported by the map even at a lower contour level (Figure 5B, second row) and the backbone peptide N atom is partly out of the map. As expected, the locally sharpened map from LocScale is less disordered with the peptide N atom at the edge of the map contour. The peptide N has a low FDR score of 0.0 compared to Cα and carbonyl C which have scores of 1.0. In this case, the backbone is likely to be misplaced as highlighted by multiple scores.

A similar case involving Leu98 is presented in Figure 5C. Leucine 98 scores low with all other metrics (Z-scores lower than −2.0) and this residue has an FDR score of 0.92. The position of carbonyl C atom is not fully supported by the map even at a lower contour and this atom has an FDR score of 0.75. In this case, multiple metrics highlight a potential backbone misfit and require further investigation to explore the possibility of improving the fit. The locally sharpened map also shows disconnected map trace at the selected contour, with the carbonyl C atom placed outside the contour. In the absence of a good reference fit for this residue, Asn109 on the other hand, is highlighted as a misfit by MapQ (0.12, Z-score −5.57), SCCC (0.49, Z-score −2.02), and PHENIX (0.58, Z-score −1.86) (Figure 5D). However, the FDR validation score assigns a value of 1.00 (Z-score 0.13) for this residue backbone, suggesting no mistrace of the backbone. A closer inspection of this position in the model shows that the lower contour level covers most of the backbone atoms of the residue, except carbonyl C atom where the map is still disconnected. The locally sharpened map is smoother with no disorder and shows a better coverage of backbone atoms. The residue is located at a low resolution area of the map and it is likely that the backbone is within the molecular volume but the atoms are misfitted, as highlighted by the other metrics.

Supplementary Figure S2C shows an area of the modeled RNA where the terminal nucleotide has a lower FDR score. The map density is also disordered at this position likely due to the higher flexibility of this part of the RNA.

### Correlation Between Different Metrics

In the cases discussed above, we show a number of cases where different metrics disagree in the detection of backbone mistrace and cases where the FDR backbone scores can be complementary. To check how different metrics rank models based on local backbone fit to maps, we scored ten of the models submitted to the 2019 Model Challenge for the target alcohol dehydrogenase map (EMD-0406), and nine of them were built using *ab-initio* model building approaches. For each model, the number of residues of chain A associated with a Z-score lower than −2.0 were counted (Table 1). The table also shows the number of residues with the FDR backbone score less than 0.9. The models in the table are sorted by the global CCC scores derived from the assessment results of the model challenge (https://model-compare.emdataresource.org/2019/cgi-bin/em_multimer_results.cgi?target_map=T0104emd_0406). No two metrics completely agree in the ranks assigned to the models based on the number of potential backbone misfits. However, there is a general agreement on best scoring the models and those with the lowest ranks. Note that the Z-scores are less meaningful in cases where the distribution of the score is far from normal. This is expected to affect the ranks, especially in the case of FSC-Q and MapQ where the outliers have significantly lower scores than the rest of the distribution.

**TABLE 1:** Outlier detection by different metrics for ten of the models submitted to the 2019 Model Challenge for the target alcohol dehydrogenase map (EMD-0406). For each model, the table number of residues of chain A associated with Z-scores lower than −2.0. For the FDR backbone score, the table also shows the number of residues with an FDR backbone score less than 0.9

ModelID	CC	Method	FDR_score <0.9	FDR_score	MapQ	SMOC	SCCC	PHENIX	FSC-Q	FSC-Q
				Z-score < −2	Z-score < −2	Z-score < −2	Z-score < −2	Z-score < −2	Z-score > 2	Z-score < −2
T0104EM035_1	0.32	*Ab-initio*	3	6	15	9	8	8	12	7
T0104EM027_1	0.32	*Ab-initio*	8	7	15	11	9	10	10	6
T0104EM010_1	0.32	*Ab-initio*	7	9	14	8	13	6	11	7
T0104EM041_1	0.32	*Ab-initio*	10	10	10	9	13	9	13	5
T0104EM090_1	0.31	*Ab-initio*	21	19	12	13	12	12	12	0
T0104EM028_1	0.31	*Ab-initio*	9	15	13	12	10	10	14	2
T0104EM025_1	0.31	Optimized	8	9	12	17	14	11	14	5
T0104EM082_1	0.31	*Ab-initio*	9	9	15	18	18	12	12	2
T0104EM060_2	0.28	*Ab-initio*	39	66	17	14	14	16	8	0
T0104EM054_1	0.27	*Ab-initio*	83	26	17	16	18	17	18	3

**FIGURE 6:**
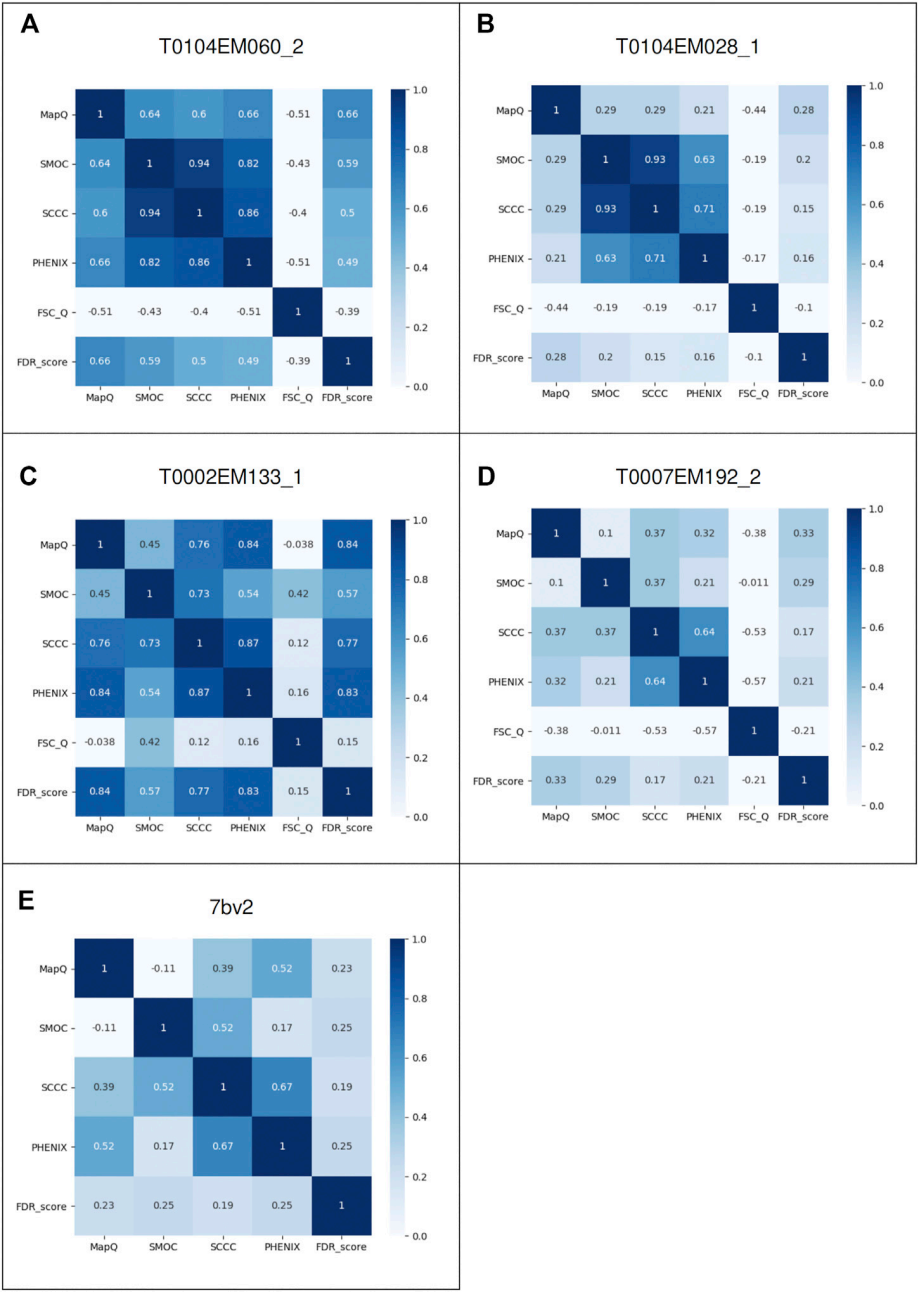
Pairwise correlations of different metrics: MapQ, SMOC, SCCC, PHENIX, FSC-Q, and FDR backbone score for the atomic models: **(A)** Chain A of alcohol dehydrogenase T0104EM060_2, **(B)** chain A of T0104EM028_1, **(C)** chain L of T20s proteasome T0002EM133_1, **(D)** chain C of γ-secretase T0007EM192_2, and **(E)** chain B of the RNA polymerase complex model 7bv2.

To further investigate the pairwise agreement between metrics, we computed pairwise correlations between scores for the case studies discussed above. Figures 6A–E present the correlation matrices highlighting pairwise correlations between metrics for each case (corresponding to Figures 1–5). In a general scenario where an atomic model fits well overall in a map but includes a few mistraced residues, the majority of the residues have FDR scores of 1.0 and we expect lower scores for mistraced residues. Hence, the FDR score being less variable relative to other scores, the pairwise correlations involving the FDR score are expected to be low. Indeed, we observe this for most of the models except for T0104EM060_2 and T0002EM133_1 where many of the residues are associated with low backbone scores (Figure 4). In these two cases, the FDR score shows better correlation with MapQ with pairwise correlation coefficients of 0.66 and 0.84, respectively. MapQ scores also correlate with SCCC and PHENIX_CC scores for these two cases.

Overall, SCCC and PHENIX CCC show a good correlation in most cases with pairwise correlations in the range 0.64 to 0.87, which is expected as both scores involve calculation of cross correlation coefficient. SCCC and SMOC scores are largely correlated as well with pairwise correlations spread between 0.37 and 0.94. These two scores use similar underlying procedures for synthetic map generation from model and identification of voxels covered by atoms. FSC-Q does not correlate with any of the other scores as the score reflects the model-map (and map-model) differences, unlike the other scores.

### Pruning *Ab-Initio* Built Models

The proposed approach was used to prune models generated by Buccaneer (Cowtan, 2006) which is an *ab-initio* model building tool that works by an iterative process involving finding backbone seed positions, growing them to fragments, connecting and pruning fragments to chains and pruning the resulting chains. Often the final model from Buccaneer needs to be pruned interactively in Coot to remove any fragments and fix any obvious mistraces. Identifying parts of the model that are fitted into low confidence regions of the map enables automated pruning of the models.

**FIGURE 7:**
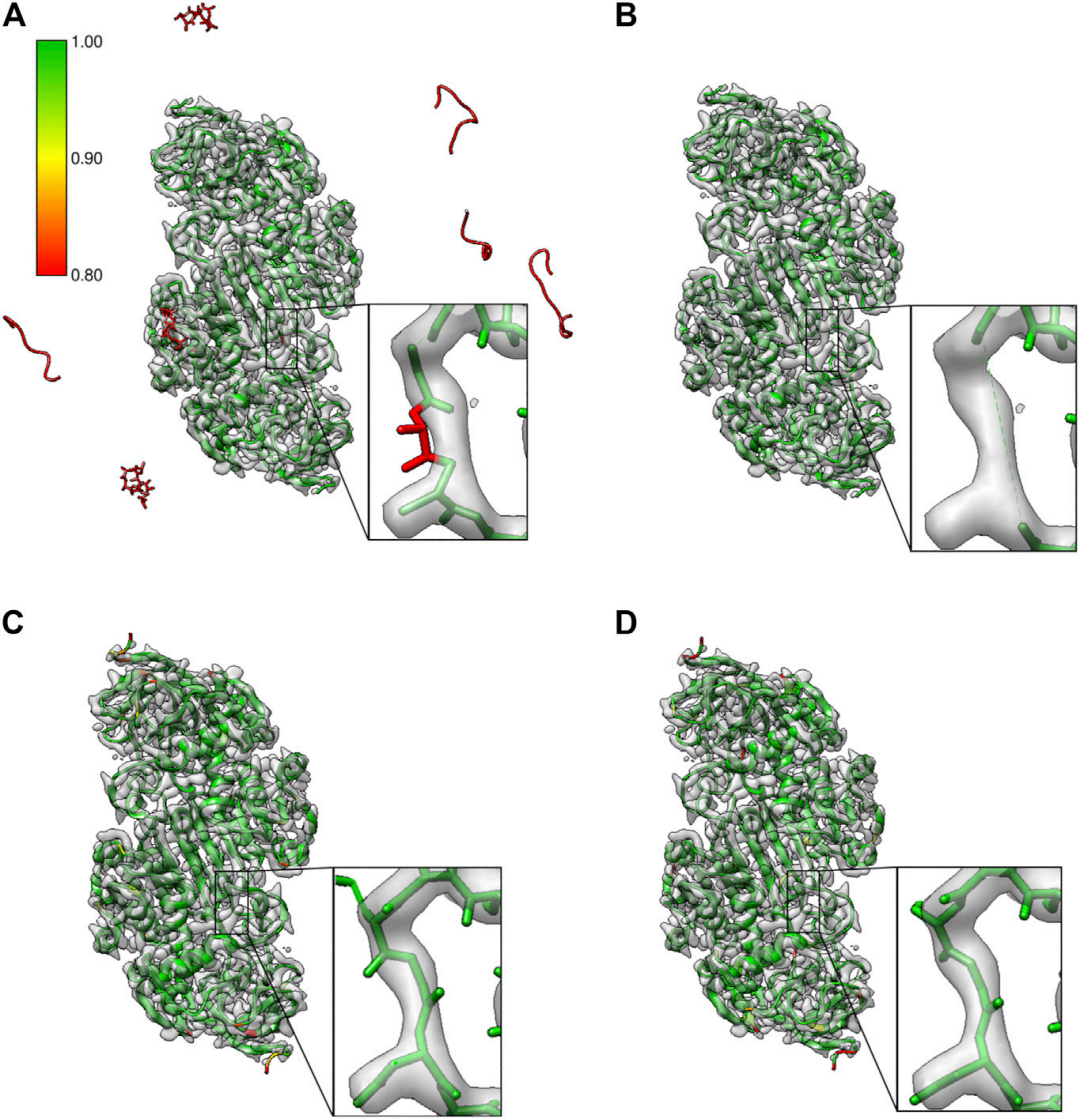
Results from pruning the model built *ab-initio* with the Buccaneer software tool on the map of alcohol dehydrogenase (EMD-0406). **(A)** *Ab-initio* built model where residues associated with low confidence scores are in red. Potential misfit of residue 107 (chain A) is highlighted, and fragments built into the background noise outside the molecular density are also associated with low confidence scores. **(B)** Model after pruning; the misfitted residue in the chain A is removed along with the preceding and following residues (Gly106-Asn108), and fragments in the background are also removed. **(C)** The model T0104EM028_1 with residues in this segment with higher confidence scores. **(D)** Reference model (PDB ID: 6nbb) with the segment highlighted.

We tested this using the *ab-initio* model built using the Buccaneer software for the 2.9 Å reconstruction of alcohol dehydrogenase (EMD-0406). Figure 7A shows the model built from four Buccaneer cycles. The confidence map–based approach identifies fragments built into the background noise outside the molecular density (highlighted in red). The zoomed area provides a closer look at the loop where one of the residues is mistraced and backbone atoms are out of the contoured map. Figure 7B shows the same model after pruning based on our approach. All the fragments and mistraced residues were removed. Residues on either side of the low scoring residue are also removed while pruning. This helps to rebuild this whole region in the next round of the automated model building. Figures 7C,D shows the confidence scores for the same segment from the model T0104EM028_1 and the reference model (6nbb.2), respectively. The residues of these models have higher confidence scores and the residues are fitted better in the contoured map.

## Discussion

The majority of cryo-EM reconstructions in EMDB are determined at resolutions worse than 3 Å and often the local resolution varies significantly in maps that are otherwise resolved at higher resolutions on an average. Hence, the chances of errors in the model are higher and validation tools that can detect errors and areas with high uncertainty, are necessary. In this study we tested an approach that evaluates the backbone trace of atomic models based on the local molecular signal (compared to background noise) in the map. The confidence scores calculated per voxel from the original map using the FDR control approach (Beckers et al., 2019) are mapped to individual backbone atoms in the model.

For the purpose of testing the approach, we used examples covering a range of reported resolutions from 2.5 to 3.4 Å. The residue backbones that have an FDR score less than 0.9 are included in Supplementary Table S1. Most of these models are built using model building and refinement tools commonly used in the field, as part of the EMDB model challenges. These challenges act as platforms to assess models derived using a wide range of modeling techniques and compare metrics which can be used to evaluate these atomic models. It also provides a repository of models built from a range of map targets and a reference model to compare against, which can be extremely useful for development and testing of new validation software.

We show that the FDR backbone score is complementary to existing model evaluation tools. The proposed score evaluates only the atomic positions and not the model agreement with the map. Hence, it is not useful for detecting any misfits within the molecular contour. Also, the current implementation of the score does not identify side chain rotamer misfits. However, as seen in many of the cases discussed in results, often backbone mistraces are associated with side chain misfits as well.

On the other hand, as demonstrated in results, some residues where one or more atoms are fitted into the background noise may still have fit-to-map scores within tolerable limits. This misplacement of backbone atoms is evident when compared to the reference, where a better backbone fit can be found. In such cases, the FDR backbone score works in a complementary manner.

Potential backbone mistraces involving a number of glycine residues were detected by the FDR backbone score (see Results), and not by other metrics. One explanation could be that glycine is often seen in flexible loops associated with low-resolution areas of the map, and some of these scores are sensitive to map resolution. In general, limiting the score calculations to backbone atoms, might also affect some of the scores like CCC, where a sufficiently large distribution of values is expected for meaningful estimation of mean and standard deviation and hence a reliable score calculation.

We also show that the approach detects weak molecular signals that are at low resolution areas of the map and not otherwise obvious. We recommend that residues associated with FDR scores less than 0.95 usually require attention and residues with scores less than 0.9 usually reflect clear cases of backbone mistrace.

We also demonstrate that the approach is useful in detecting residue mistraces in a model. Hence, the tool is useful as part of iterative model building pipelines or to evaluate the final model. Automated pruning of models based on this approach can be a useful step in the iterative model building and refinement process. Models after pruning can be also a starting point for extending or iterative building with the Buccaneer model building tool. As presented in the results, the approach is useful to validate ligands, carbohydrates, and nucleic acids as well.

**FIGURE 8:**
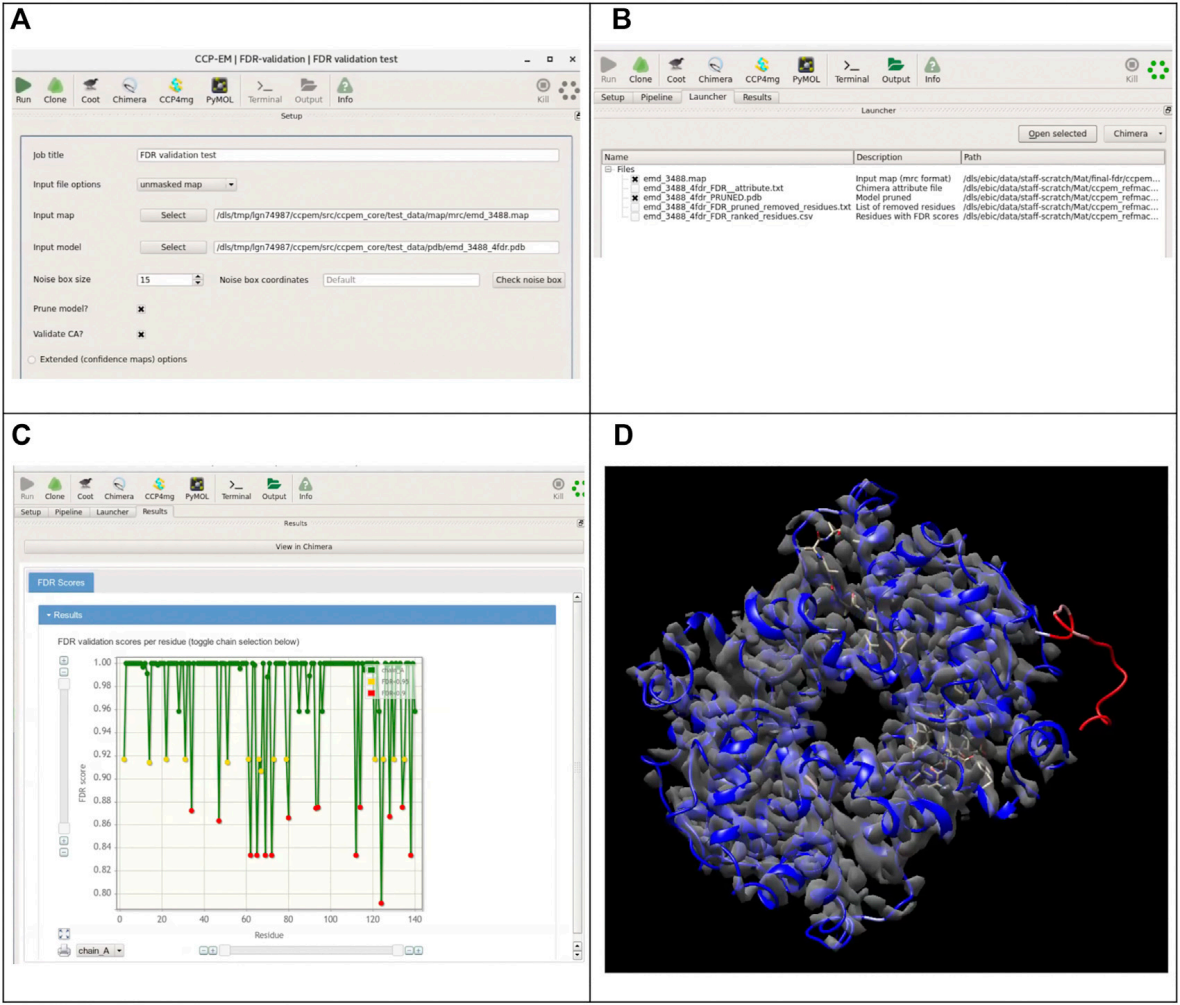
Integration of the presented tool with the CCP-EM software suite. **(A)** Input interface for the FDR-validation task. The original map (preferably unmasked) or a precalculated confidence map and a model are required as inputs. If the original map is provided as input, a confidence map will be calculated internally. Users have access to the advanced setup options for confidence map job. **(B)** Overview of the launcher tab listing the output files. **(C)** Results tab with the FDR backbone scores plots for each chain. **(D)** Resulting models colored according to calculated confidence score, overlaid with the confidence map in UCSF Chimera (accessible from the results tab of the CCP-EM task).

The implementation of this score as a tool in the CCP-EM software suite makes it easily accessible for the cryo-EM community. The described software tool is available from the CCP-EM suite as “FDR validation task” confidence map calculation can be run as part of this task, where the user has to provide the original map (preferably unmasked) and the model to validate. As an option, the user can adjust the size of the noise box used for calculation of background statistics. In some cases, especially if the specimen is significantly elongated in one direction, users should also check the preview of the noise boxes to make sure that the noise box does not contain any part of the molecular volume. The extended options for the confidence maps section allows to set the advanced parameters for the FDR maps calculation (Figure 8A).

If the user has already generated the FDR map, it can be used directly as an input (Figure 8B). Instead of a confidence map, any custom map can be used as well and residues will be assigned scores based on values in the map. Validation based on the scores of backbone atoms is run by default, users can additionally choose to validate only the CA positions. Optionally, the model can be pruned further to remove residues associated with low confidence scores. A model file with atomic b-factors replaced by the confidence scores and a CSV file containing the confidence scores for each residue are generated as outputs. If the option to prune the model was chosen, a pruned model is provided as the additional output, along with a text file containing the list of all removed residues. Figure 8C shows the launcher tab with a list of output files generated from the job. On the results tab, a link is included to open the resulting models directly in UCSF Chimera with the model colored based on the confidence scores. Figure 8D shows the results open directly in UCSF Chimera with the resulting model colored according to the confidence score.

For a confidence map of size 192 × 192 × 192 voxels, the assignment of FDR scores to residues takes about 0.22 s on a PC with specification: Intel(R) Core(TM) i5-8250U CPU @ 1.60 GHz x 8, 8 GB RAM. The latest CCP-EM nightly release available from https://www.ccpem.ac.uk/download.php includes the implementation of “FDR validation task” The source code of the tool for evaluating atomic models based on confidence maps is available from (https://github.com/m-olek/FDR-validation).

## Conclusion

In this study, we present a tool for validating atomic models derived from cryoEM maps. It works based on the calculation of the confidence maps, which estimates molecular signal to noise at every voxel, and also detects weak signals from the low resolution areas of the map. This helps to assess atomic positions based on the local information in the map and identify mistraced residues in the model. This approach is complementary to other validation tools that quantify agreement with the map, as it evaluates atom positions based on the local map information. We believe that, with the integration with the CCP-EM software suite, the presented tool will be a useful addition to the existing validation tools.

## Data Availability

The raw data supporting the conclusions of this article will be made available by the authors, without undue reservation.
