# Supplementary material for: Cryo-EM Map–Based Model Validation Using the False Discovery Rate Approach
Source: Front Mol Biosci. 2021 May 18;8:652530. doi: 10.3389/fmolb.2021.652530 (PMC8167059; doi:10.3389/fmolb.2021.652530)
Supplement: Supplementary file 1 [file Table1.DOCX]

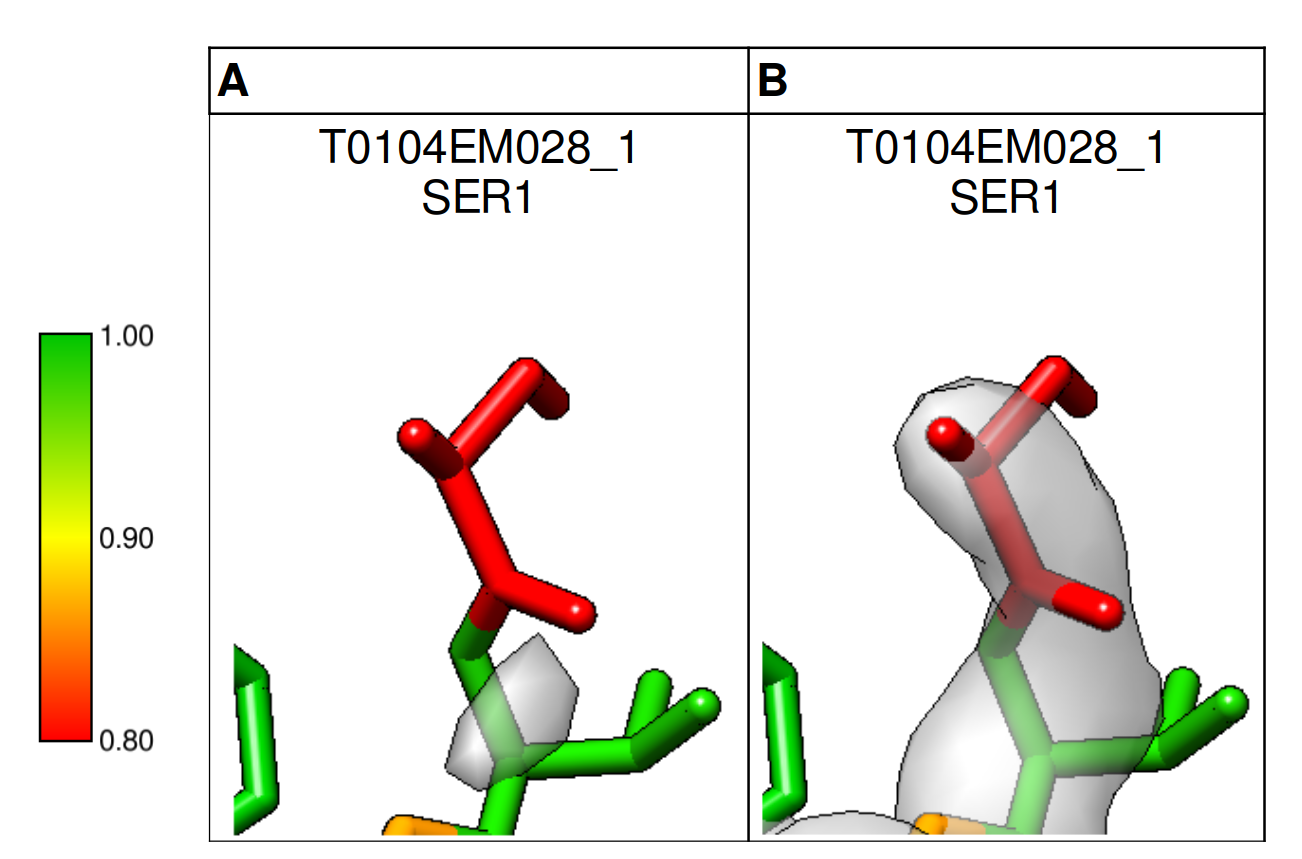


**Supplementary Figure 1.** FDR backbone score for the Chain A of the atomic model T0104EM028_1 submitted to the EMDB model challenge for the target alcohol dehydrogenase map (EMD-0406). Ser1 is shown fitted in the map rendered at the recommended contour level of 0.02 (A) and a lower contour level of 0.013 (B).


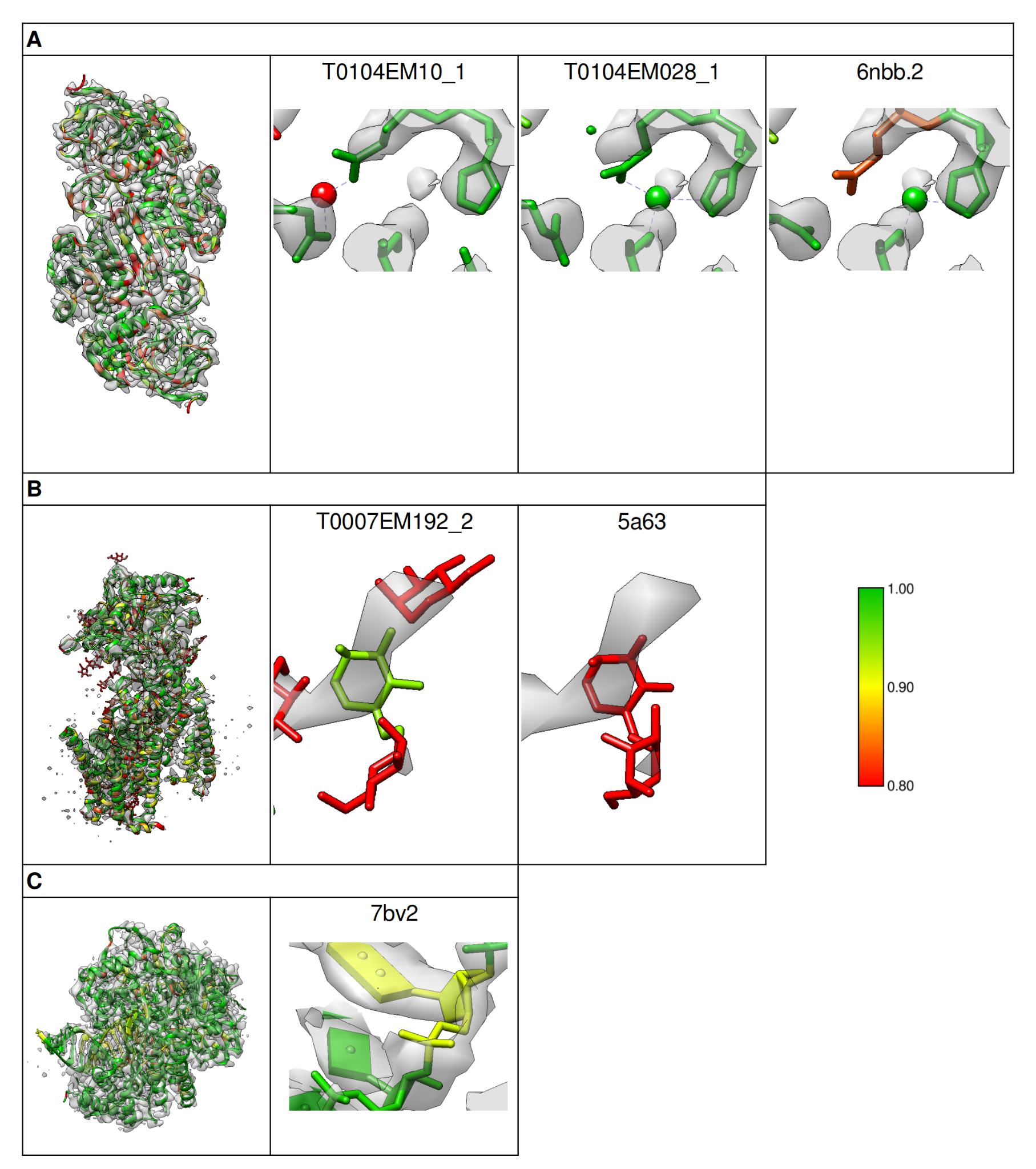


**Supplementary Figure 2**. FDR backbone validation for ligands and nucleic acids, for (A) atomic models submitted to the EMDB Model Challenge for target map of alcohol dehydrogenase (T0104). Left panel: model T0104EM010_1 where the zinc ion has a low confidence score; Central panel: atomic model T0104EM028_1,where the zinc ion (403, chain A) position has a high confidence score; Right panel: the zinc ion in the reference model 6nbb associated with a high confidence. (B) atomic models submitted to the EMDB Model Challenge for target map of γ-secretase. Left panel: polysaccharide chain terminal associated with lower confidence scores (NAG 1716- BMA 1718, chain A); Right panel: partial polysaccharide model from the reference structure (PDB ID: 5a63). (C) atomic model of the RNA polymerase complex (PDB ID: 7bv2) showing the terminal base (U8, chain T) of the RNA model associated with lower confidence scores.
